# Provision of Interventional Radiology Services 2023

**DOI:** 10.1007/s00270-023-03600-0

**Published:** 2023-11-17

**Authors:** Robert Morgan, Philip Haslam, Ian McCafferty, Timothy Bryant, Christopher Clarke, Simon McPherson, David Wells, Yuri Gupta, Teik Choon See, Raghu Lakshminarayan, Fiona Miller, Paul Scott, Bahir Almazedi, Harry Bardgett, Alex Barnacle, Nadeem Shaida, Dinesh Manoharan, Mark Lewis, Jeremy Taylor, Rajesh Bhat, Behnam Shaygi, Lakshmi Ratnam

**Affiliations:** 1 St George’s, University of London, London, UK; 2 Freeman Hospital NHS Trust, Newcastle Upon Tyne, UK; 3 Birmingham Hospitals, Birmingham, UK; 4 University Hospitals Southampton NHS Foundation Trust, Southampton, UK; 5 Nottingham University Hospitals NHS Trust, Nottingham, UK; 6 Leeds General Infirmary, Leeds, UK; 7 University Hospitals of the North Midlands NHS Trust, Stoke-On-Trent, UK; 8 University Hospitals Sussex NHS Foundation Trust, Brighton, UK; 9 Cambridge University Hospitals NHS Foundation Trust, Cambridge, UK; 10 Hull University Teaching Hospitals NHS Trust, Hull, UK; 11 King’s College Hospital NHS Trust, London, UK; 12 Hull University Teaching Hospitals, Hull, UK; 13 York Teaching Hospital, York, UK; 14 Bradford Teaching Hospitals, Bradford, UK; 15 Great Ormond Street Hospital for Children, London, UK; 16 Cambridge University NHS Foundation Trust, Cambridge, UK; 17 Sheffield Teaching Hospitals NHS Foundation Trust, Sheffield, UK; 18 Norfolk and Norwich University Hospitals NHS Trust, Norwich, UK; 19 Frimley Health Foundation Trust, Surrey, Frimley UK; 20 Ninewells Hospital and Medical School, Dundee, UK; 21 London North West University Healthcare NHS Trust, London, UK

**Keywords:** Interventional radiology, Clinical practice, Vascular, Non vascular

## EXECUTIVE SUMMARY

Interventional radiology is a key service in all hospitals in the UK. Interventional radiology units vary in size depending on the size of the hospital, the services provided by the hospital and their proximity to other units. All patients should have access to emergency interventional radiology procedures whenever they require them. This will require a network arrangement between hospitals in some cases. An out-of-hours interventional radiology on-call rota of at least one in six is the minimum standard that should exist.

A wide number of clinical conditions are treated by interventional radiologists and the number of procedures grows year on year. Some basic interventional radiology procedures can also be performed by appropriately trained allied health professionals.

Interventional radiologists should aspire to take primary responsibility for their patients and should adopt all tenets of Clinical Practice to improve outcomes and patient safety. Interventional radiologists should work with clinical colleagues to always provide optimal care for patients.

Although interventional radiology is a subspecialty of radiology under the umbrella of the Royal College of Radiologists (RCR), this arrangement does not provide all of the needs required by interventional radiology and interventional radiologists. A change of this model to an interventional radiology specialty or a separate IR faculty within the RCR would provide substantial benefits for interventional radiology, not least by establishing the autonomy to increase the IR workforce to meet the demands of patients.

Interventional radiology is examined along with diagnostic radiology in the RCR examinations at the end of specialty training year three (ST3). After that, there is no test of knowledge or aptitude for interventional radiologists before they start to practise as consultants. Similar to surgical specialties, there should be a specific examination in interventional radiology to assess competency before commencing a consultant post.

Audit and registries are an essential component of professional practice. All interventional radiologists should submit data on their peripheral vascular procedures to the UK National Vascular Registry. There is currently no national registry of interventional radiology procedures. The British Society of Interventional Radiology (BSIR) aspires to institute such a registry, but issues with funding prevent further progress. Further work is required with funding agencies to take this forward.

Paediatric interventional radiology is an important area of IR that is generally not adequately provided for in the UK, both in terms of specialist IRs with a focus on paediatric IR and also in terms of general IRs who will perform IR procedures on young patients. Allied to this is a relative lack of anaesthetic provision, which is key to the provision of a sustainable paediatric IR service in the UK. This situation must be rectified to address the increasing demand for paediatric IR in the UK.

In addition to the lack of anaesthetic provision for paediatric IR, the lack of anaesthetic provision for general anaesthesia and deep sedation for adult IR procedures is also a challenge in many departments.

Finally, research and innovation are essential for the development of new treatments, and also for the assessment of existing ones to assess their efficacy in comparison with other therapeutic options. Interventional radiologists should engage in research wherever feasible, encourage a research ethos in trainees under their tutelage, and support the institution of increased designated academic interventional radiology positions nationally.

## CONTENTS

Authors

Introduction

What is Interventional Radiology (IR)

Who Performs Interventional Radiology Procedures?

The Interventional Radiology Team

The Interventional Radiology Theatre

Provision of IR: Location

Provision of IR: Timing

Clinical Practice

Training

Accreditation for IRs

Accreditation for IR Centres

Governance

Major Clinical Indications for Interventional Radiology

Paediatric Interventional Radiology

Women in IR

Informing Patients About IR Procedures

Turf Issues

Subspecialty and Specialty Status

Research and Innovation

Burnout

Summary

## INTRODUCTION

This is the third edition of the UK Provision of Interventional Radiology Services (POIRS) document. Significant change has occurred since the second edition of POIRS was published as a joint document by the British Society of Interventional Radiology (BSIR) and the Royal College of Radiologists (RCR) in 2019. In response to ongoing developments in Interventional Radiology practice, this edition has been expanded to include a more comprehensive discussion of Clinical Practice, key IR procedures, paediatric IR, women in IR, research, and registries.

A call to the BSIR membership in late 2022 invited expressions of interest from members who might be interested in contributing to this third edition of POIRS. This document has been created from the author-submitted sections by Professor Robert Morgan, Doctor Philip Haslam and Professor Ian McCafferty.

## WHAT IS INTERVENTIONAL RADIOLOGY?

Interventional Radiology is a discipline that performs procedures using minimally invasive percutaneous techniques under imaging guidance to treat patients (1). Interventional radiology procedures are performed in all hospitals in the United Kingdom (UK) by radiologists trained in interventional radiology techniques. Interventional radiology is a subspecialty of radiology recognised by the General Medical Council (GMC). The majority of IR procedures are therapeutic in nature with interventional radiology having a potential role in all body systems. Interventional radiologists are not just technologists. Interventional radiologists must regard themselves as clinicians and see patients in clinic settings to discuss procedures with them following referral, and at follow-up clinic visits after the procedures have taken place. Interventional radiologists have a responsibility to engage in research to further the boundaries of IR and to educate trainees, other interventional radiologists, diagnostic radiologists, clinical colleagues, and the general public regarding the benefits of interventional radiology in UK medical practice.

**Reference**

2.1: Adam A. The definition of interventional radiology (or “when is a barium enema an interventional procedure?”). Eur Radiol 1998 8: 1014–1015

## TYPES OF INTERVENTIONAL RADIOLOGIST

There are several types of interventional radiologist. These may vary with the extent of time that they devote to interventional radiology versus diagnostic radiology, and their scope of IR practice. As a rule, to be classified as a dedicated interventional radiologist, over 50% of their workload is likely to be IR-specific (including procedural work, clinics, and administration). Most interventional radiologists will also have diagnostic duties, some of them specialist and related to their area of interest, others more general work.

In general, interventional radiologists can be divided into Specialist IRs, General IRs, and Diagnostic Radiologists (DRs) that provide interventional procedures within the remit of their specialist area. Inevitably, the distinction between the three types of IR is vague and artificial. The broad categories are described below.

### Specialist IR

These individuals tend to work in larger hospitals, usually alongside specialist clinical services, in sizeable IR departments (usually with at least 6 IRs). These hospitals include major trauma centres, gastrointestinal haemorrhage centres, vascular surgical centres, tertiary oncological and surgical centres, transplant units, specialist paediatric hospitals and neurosurgical centres. Within these specialist centres, more complex interventions are likely to be undertaken. There is often subspecialisation within these IR departments along multidisciplinary team (MDT) lines. Procedures performed may include complex peripheral and arterial interventions, endovascular aortic interventions (including thoracic aortic repair, fenestrated and branched aortic repair); interventional oncology procedures such as embolisation (including chemoembolisation and radioembolisation) and tumour ablation; transjugular intrahepatic portosystemic shunt insertion, complex biliary and uroradiological interventions, uterine and prostate artery embolisation, vascular and lymphatic malformation work, paediatric intervention and neuro-interventions including stroke thrombectomy. There would likely be a significant volume of acute and emergency work passing through these centres requiring on-site provision of 24/7 IR cover.

### General IR

These individuals tend to work in smaller centres and district general hospitals often as part of a wider network group as part of a hub and spoke arrangement. Some of these individuals may also provide certain specialist services at a hub hospital. Clinical provision would include all aspects of interventional radiology to support allied clinical services where specialist intervention and management is not indicated. This should include all essentials of IR management including urgent/emergency work (management of acute haemorrhage, renal and biliary obstruction), alongside elective work (peripheral vascular intervention, transarterial embolisation (including uterine artery embolisation in the presence of adequate gynaecological support). The scope of services provided would depend on the level of clinical support on site and will vary from hospital to hospital. Some smaller hospitals also have their own specialist areas/units requiring more complex and specialist interventions that could either be provided by the local ‘general’ IR, or by support from the hub. There is likely to be less requirement in terms of cases for emergency out-of-hours procedures making the provision of 24/7 cover challenging. How hospitals meet these challenges is discussed below.

### Diagnostic Radiologist Who does Some IR Procedures

The variation within this group is considerable. This could encompass a subspecialist DR in a tertiary unit who provides limited specialist intervention. Examples would include a DR contributing to or providing a tumour ablation service (e.g. microwave ablation within the liver or renal cryoablation); a gastrointestinal (GI) radiologist who also performs endoscopic ultrasound, percutaneous biliary drainage or gastrostomy insertion; a genitourinary radiologist inserting nephrostomies and supporting the percutaneous nephrolithectomy service. This can be a safe and sustainable solution in certain areas and can be driven by local demand. It is important that the individuals involved are properly trained, have enough exposure to maintain those skills and are not operating as single-handed practitioners (which would be a service risk).

### Other

In addition to interventional radiologists, there is also an increasing number of allied health professionals who are trained to provide aspects of interventional radiology. These include advanced radiographic practitioners, clinical nurse specialists and physician associates (see below).

## THE INTERVENTIONAL RADIOLOGY TEAM

There are several members of the interventional radiology team.

### Consultant Interventional Radiologists

Skilled specialist doctors that provide minimally invasive image-guided therapies and perform IR procedures, and work together with different team members to develop and deliver IR services within a hospital or region. They lead or provide input into MDT discussions as part of a wider therapeutic and diagnostic team.

### Interventional Radiology Trainees

Subspecialty trainees who work closely with consultant interventional radiologists to learn the clinical and team-working skills, knowledge, and behaviours to become future consultants as outlined in the interventional radiology curriculum. Some may start IR training from an early stage of clinical radiology training, and others may elect to subspecialise in IR midway through clinical radiology training.

### Clinical Radiology Trainees

Clinical radiology trainees will rotate through interventional radiology as part of their core radiology training (years 1–3). These trainees should experience a broad range of IR procedures and training in some basic procedures as outlined in the RCR curriculum. These may include core radiology procedures such as percutaneous biopsy and percutaneous drainage of fluid collections.

### Radiographers

Interventional radiographers have detailed knowledge on the safe and appropriate use of ionising radiation as well as the imaging equipment and range of interventional procedures. They ensure that optimal quality images are obtained whilst keeping radiation doses as low as reasonably practicable.

Interventional radiographers are also key members of the IR team for many IR procedures, particularly when advanced imaging techniques are required, e.g. for interventional oncology procedures. For these latter techniques, additional training of interventional radiographers is required. Ideally, this would be provided by the Society of Radiographers during radiographic training. However, training is usually delivered locally depending on the specific requirements of each IR centre.

### Nurses

Interventional nurses are essential to support the interventional radiology team as they may assist with interventional procedures, assess the patient before and after their procedure, are familiar with the IR equipment and theatre and are trained to give intravenous (IV) sedation when required.

### Administrative Personnel

They perform the majority of the administrative tasks to support the smooth and efficient running of the interventional radiology service including alerting teams to urgent and pending appointments, booking clinic and theatre appointments, communication with patients, facilitating referral between different specialties and supporting multidisciplinary team meetings (MDTs), morbidity and mortality meetings (e.g. REALM) and other governance activities. They are also the most aware of the targets required of the interventional radiology service and are well placed to identify potential delays to patient care. Management of the IR device stock is also usually the responsibility of the administrative staff, who may be assisted by nursing or radiographic staff members.

### Advanced Practitioners (AP), Clinical Nurse Specialists (CNS)

APs and CNSs are healthcare professionals, typically nurses or radiographers, trained to further support the IR service. This may include peri-procedural care or ward outreach work, performing interventional procedures independently, or undertaking IR research.

### Physician Associates (PA)

PAs are healthcare professionals who work alongside doctors to provide medical care. There are currently only a few IR PAs in the UK. They work within a defined scope of practice determined locally and are currently under control of the GMC. PAs are not able to prescribe or request ionising radiation in the UK.

## THE INTERVENTIONAL RADIOLOGY THEATRE (IRT)

Interventional radiology procedures may be performed in a variety of locations depending on the procedure involved. Potential locations include dedicated theatres with state-of-the-art angiography equipment, fluoroscopy rooms, treatment rooms with an ultrasound machine, CT scanners and MRI scanners.

A requirement for any department wishing to perform angiography, embolisation or complex non-vascular procedures is an interventional radiology theatre (IRT) that contains state-of-the-art angiography equipment. Ideally the IRT should be located within imaging with access to anaesthetics, whilst this is not essential, it allows interventional radiologists access to all specialised imaging.

### Interventional Radiology Theatre: Overview of Requirements

All hospitals delivering emergency, trauma, surgical or obstetric services should ensure prompt access to one or more appropriately equipped IRT. The IR department must include a defined patient reception area, a patient preparation area, and a post-procedure recovery area. In some circumstances, the patient preparation and recovery areas will be combined. Each IRT should be at least 60m2, or 75m2 for a hybrid theatre suite (1). Endovascular aortic aneurysm (EVAR) procedures should not be performed using mobile angiography units (2). Patient preparation, intervention and recovery areas must allow unimpeded patient flows for day-case, in-patient and emergency cases. Input from interventional radiologists is essential when planning new, or changing existing facilities, to support effective patient care pathways.

Strict adherence to end-of-life equipment replacement programmes is essential for patient and staff safety. IR operations continue to increase in volume, complexity and novelty. IR facilities planning must allow for future expansion. It is recommended that the suitability of the IR estate and equipment is reviewed on a periodic basis—and at least every five years. Financial constraints must not be used to limit facilities below the following standards.

### Angiography Equipment for the IR Theatre

- Ceiling or floor mounted fixed angiographic equipment with a large field of view (e.g. 48cm), multi-obliquity imaging, road mapping, acquisition overlay facility and a radiation protected control bay are minimum facilities.
- Radiation shielding (table, ceiling and floor mounted, fixed or mobile) should be effective in all positions of the C-arm. Floor marking encourages radiation safety.
- Automated contrast-pump injectors and ultrasound are standard.
- Desirable adjuncts include a variable rotation image intensifier, rotational angiography (on-table CT, 3D angiography) and fusion imaging.
- Biplanar systems are required for neurovascular interventions.

### Essential Facilities in the IR Theatre

- Access to diagnostic imaging on PACS and electronic patient records (EPR).
- IT solutions for the reporting & recording of the interventional procedure (operation notes) with post-intervention advice should be available to the ward immediately after the procedure. The interventional procedure notes should be seamlessly transferred to all relevant electronic systems, e.g. radiology information system (RIS) and electronic patient record (EPR).
- Complex IR operations require access to elective and emergency anaesthetic support. Anaesthetic equipment and gases supply must allow for all necessary angiographic equipment and patient positions. The anaesthetic equipment, including a difficult airway trolley, should be checked with the same frequency as open surgery theatres.
- Lighting suitable for combined IR and surgical operations.
- IRT positive pressure ventilation at 10 air changes/hour (ac/hr), increasing to 15 ac/hr if anaesthetic gases are used. Hybrid theatres require 22 ac/hr [3].
- Angiographic uninterrupted emergency electrical power.
- Separate scrub and gowning facilities for at least two individuals.
- Locked drug cabinets (including anaesthetic).
- Routinely used angiographic consumables and emergency equipment such as stent grafts, occlusion balloons and embolic agents may be stored in the IRT or in a storage room very close to the IRT.
- Paediatric patients require a dedicated equipment inventory.

### Support for the IR Theatre

- Dedicated day-case facilities (in or adjacent to the IRT) can reduce costs, improve patient throughput and overall efficiency.
- Access to level 2 and 3 beds is often required for elective and emergency cases.
- Protocols to ensure rapid access to surgical and medical support for complications are essential.
- Anaesthetic support to enable selected procedures to be performed under general anaesthesia or deep sedation.

### Anaesthetic Support

The assistance of the anaesthetic department in each hospital is essential to enable IRs to be able to perform procedures on patients who require general anaesthesia or deep sedation. These may include transjugular intrahepatic portosystemic shunts, percutaneous biliary procedures, and any patient unable to tolerate procedures under light sedation. Collaboration with the anaesthetic department is important to facilitate these procedures. Some hospitals have dedicated anaesthetists who perform one or more lists with their IR colleagues a week. However, this type of anaesthetic cover is not a widespread phenomenon. More commonly, there is a lack of anaesthetic support for IR departments. This impacts negatively on patient experience, patient throughput and scheduling, which prolongs patient waiting times before procedure booking or their bed occupancy if they are inpatients. There is a general need in the UK for improved provision of anaesthetic services for interventional radiology.

**References**

5.1.: Mark O. Baerlocher, Sean A. Kennedy, Thomas J. Ward, Boris Nikolic, Curtis W. Bakal, Curtis A. Lewis, JD, Adam B. Winick, Gerald A. Niedzwiecki, Ziv J Haskal, and Alan H. Matsumoto. Society of Interventional Radiology: Resource and Environment Recommended Standards for IR. J Vasc Interv Radiol 2017; 28:513–516 10.1016/j.jvir.2016.12.1213
5.2.: Provision of Interventional Radiology Services. Second edition 2019. Royal College of Radiologists. https://www.bsir.org/media/resources/provision-interventional-radiology-services-second-ed2019_klzYwZt.pdf
5.3.: Health Technical Memorandum 03-01 Specialised ventilation for healthcare premises Part A: The concept, design, specification, installation and acceptance testing of healthcare ventilation systems (2021) https://www.england.nhs.uk/wp-content/uploads/2021/05/HTM0301-PartAaccessible-F6.pdf

## PROVISION OF IR: LOCATION

The concept of an interventional radiology Hub and Spoke Network to deliver comprehensive in- and out-of-hours care for all patients across a geographical region emulates the reconfiguration of national vascular services over the last decade (1). The need for such a model to improve access to IR services was highlighted in the 2020 UK Radiology GIRFT report (2), which found that around 60% of trusts do not provide a 24/7 in-house nephrostomy and embolisation service, and many lack robust pathways for transferring these patients elsewhere.

An IR Network can be defined as a group of hospitals within a defined geographical area, consisting of a single hub hospital and several spoke hospitals, which have agreed pathways for the care of elective and emergency patients from across the region*.* The hub serves as the loco-regional centre for complex IR procedures in and out of hours, while non-complex and day case procedures are delivered at both the hub and the spoke hospitals (3).

The hub should employ a minimum number of IR Consultants (at least six) and always have consultant presence on site in normal working hours. Daytime services will include day case and inpatient complex vascular and non-vascular procedures. The hub must provide a 24/7 out-of-hours on-call rota for haemorrhage and sepsis control, with a clearly defined scope of procedures undertaken out-of-hours, and robust agreed pathways to allow transfer of patients from across the network. The IR hub is likely to be co-located with other specialties’ network hubs (e.g. the vascular hub, the major trauma centre).

Spoke hospitals may employ fewer IR Consultants (rotating out from the hub or based solely at the spoke site), who should ideally participate in the network on call rota. There may be less than a five-day IR consultant presence and usually no dedicated out-of-hours in-house IR service is provided. Straightforward day case and inpatient referred procedures are undertaken (e.g. femoral angioplasty, nephrostomy). No complex or hybrid cases (e.g. EVAR) or those that require post-procedure admission (e.g. RIG) are provided at the spoke hospitals.

**References**

6.1: Vascular Surgery, GIRFT Programme National Specialty Report, March 2018.
6.2: Radiology, GIRFT Programme National Specialty Report, November 2020.
6.3: BSIR Provision of Interventional Radiology Services, 2nd Edition.

## PROVISION OF IR: TIMING

Interventional radiology procedures are performed as scheduled non-emergent procedures (i.e. elective), scheduled urgent procedures or as emergency procedures. Elective and urgent procedures are usually scheduled within normal working hours, while emergency procedures may be performed within normal hours or out-of-hours depending on when they arise.

### Elective Service Provision

Many patients can undergo their elective IR procedure with admission and discharge on the same day, i.e. day case procedures. Other patients requiring more complex procedures, or who have significant comorbidity, may require their procedure while in a hospital bed, either after or before and after their procedure. Patients requiring urgent non-emergent procedures are usually already in hospital.

### Day Case IR Provision

One of the key benefits of interventional radiology is the ability to deliver ambulatory (day case) care. There is accruing evidence that illustrates how this can be achieved successfully (1–3). In 2023, the provision of dedicated facilities for day case procedures is variable across the UK. In units where dedicated IR day case facilities are not available, the ability to offer patients their procedures as a day case is limited and depends on the availability of beds in surgical day case units or on the wards. This has the additional drawback that patients housed in these latter areas are cared for by nursing staff who may not have specific knowledge or understanding of IR procedures and their potential complications. This may lead to misunderstandings and in some circumstances unsafe practice.

The optimal standard of care is the provision of a radiology day case unit (RDCU) in each hospital. This should be staffed by nurses that rotate through IR clinics, IR theatres and the RDCU, providing comprehensive IR care from admission to discharge. In some centres, there are barriers to this ideal, including a lack of physical space, staff recruitment and retention, and funding. However, lessons learned during the Covid pandemic showed how day case units can keep the IR department functioning and can provide major benefits for patients. A business case template illustrating the benefits of a dedicated RDCU can be found on the BSIR website (4).

### Access to Inpatient Beds

Not all elective patients can be treated as day cases. Some elective procedures require admission of the patient for an overnight stay for monitoring purposes after their procedure; and others require admission the day before the procedure. For the scheduling of these more complex elective procedures to run smoothly, interventional radiologists should have straightforward access to inpatient beds to accommodate IR patients either side of their procedures where required. The case for access to inpatient beds was made in a recent publication that was produced by a BSIR Task Force (5).

### Out of Hours IR Provision

There are a number of models that are used to provide out of hours (OOH) IR procedures within and across Trusts. For reasons of patient and staff safety, the minimum on-call rota that IRs should be required to work is a one in six rota.

Interventional radiology services with fewer than six interventional radiologists should liaise with neighbouring units to develop models of care that will permit robust and workable IR rotas across sites. Some centres with limited numbers of their own interventional radiologists on site may provide OOH IR cover in a multi-trust IR Network. IRs may arrange to travel between sites in the network to perform OOH IR procedures at the specific site where the patient is located. Alternatively, all OOH procedures may be performed at a single site, i.e. hub. Patients requiring an OOH procedure may need to be transferred from the spoke hospital to the hub hospital for their IR procedure.

The ideal model is that the centre, or group of centres, runs a dedicated IR on-call rota separate to the diagnostic rota, which provides emergency cover for core IR procedures, such as the drainage of obstructed kidneys and embolisation of haemorrhage. The core procedures must be performed by all of the IRs on the on-call rota. Other IR procedures may be provided OOH according to local IR expertise and agreement.

Some larger centres choose to split aspects of IR and run parallel OOH rotas, such as non-vascular and vascular IR, although these latter types of practice require an overall large number of IRs to make them workable and adhere to a one-in-six minimum rota. Most departments do not have enough IR consultants to provide this type of rota. In general, it is more straightforward for all IRs to be able to perform the core OOH procedures outlined above than to form separate rotas along individual subspecialist interest lines.

It is essential for good safe medical care that these across-site OOH relationships are detailed with service level agreements (SLA’s) to provide funding for capital costs, recurring costs for equipment, staffing and disposable items along with clear written pathways for inter-hospital patient transfer.

In general, network cover, while effective in some regions, fails to provide optimal OOH IR cover in many other locations. There is an ongoing need to by each integrated care system (ICS) in the UK to organise and provide robust OOH IR cover for all patients in that ICS. Network arrangements for IR OOH cover are facilitated by joint IR consultant appointments between trusts in each ICS with the consultant on call for the hub unit itself (6, 7).

### Recommended Rotas

As stated above, the minimum OOH rota should be 1 in 6. For larger busier trusts and some IR networks, especially where there is provision of major trauma services and specialist aortic work, a minimum rota should be 1 in 8 interventional radiologists, because of higher volumes of IR work out of hours.

**References**

7.1.: R. Lakshminarayan, C. Bent, J. Taylor, T. Bryant, R. Ahmad, A. Diamantopoulos, R.A. Morgan Developing day-case units: imperative for optimal patient care in interventional radiology Clin Radiol. 2023; 78: 295–300. 10.1016/j.crad.2022.11.017
7.2: Wells R.D. Ambulatory care in interventional radiology: a framework for radiology day-case. *Clin Radiol.* 2022; 77: 489–495. 10.1016/j.crad.2022.03.010
7.3: Roson N, Antolin A, Mast R, et al. Experience and results after the implementation of a radiology day unit in a reference hospital. *Insights Imaging.* 2022; 13: 109
7.4: BSIR. Business case template. https://www.bsir.org/static/uploads/resources/BSIR_TASKFORCE_DAY_CASE_BUSINESS_CASE_TEMPLATE.pdf
7.5: Bryant T, Diamantopoulos A, Ahmad R, Lakshminarayan L, Bent C, Taylor J, Morgan R. Access to beds for interventional radiology patients – improving patient care. Clinical Radiology 2023. 10.1016/j.crad.2022.10.016
7.6: Morgan R. Out of hours provision for interventional radiology services in London. Current status, gaps and needs. NHS England 2023.
7.7.: https://www.england.nhs.uk/improvement-hub/wp-content/uploads/sites/44/2017/11/Seven-Day-Access-to-Interventional-Radiology.pdf2.

## CLINICAL PRACTICE

Full integration of interventional radiology practice into standard patient care requires ownership of the IR service in conjunction with a safe, effective, and sustainable clinical practice. Interventional radiologists must assume primary clinical responsibility for their patients in the same way as other clinicians. Moreover, IRs should participate on an equal basis in the decision-making process, e.g. on how a particular cancer should be treated.

A good clinical IR practice also involves having a system in place for assessing the patient before the procedure; and follow-up at a time after the procedure to assess the outcome for the patient. The success of IR practice requires interventional radiologists to take responsibility for the patient throughout the entire clinical process (1).

### Pre-procedural Assessment

A full evaluation of the indication, suitability, and best approach for the procedure should be completed before the intervention. This involves a multidisciplinary team (MDT) consensus with the referral teams and other clinical disciplines, e.g. surgeons, physicians, oncologists, and palliative teams where relevant. In addition to imaging evaluation, the patient’s performance status, medications (particularly antiplatelet and anticoagulant agents), and blood parameters should be reviewed (2). It is also important to establish what imaging modality is best suited to guide the intervention and whether the procedure can be performed under sedation and local anaesthesia, or if general anaesthesia is required. The outcome of the MDT discussion should be documented in the patient’s medical record.

### Interventional Radiology Clinic

Ideally, all patients referred for an interventional radiology procedure should be assessed in an interventional radiology clinic. A lack of workforce locally may necessitate that only referrals for the more complex procedures (e.g. lower limb angioplasty, uterine artery embolisation) are seen in the IR clinic. However, wherever possible, IRs must make every effort to institute a pre-procedure assessment clinic in their hospital.

Interventional radiology clinics are one of the most important steps to establishing a full clinical IR service. The main aim of an IR clinic is to gain a full understanding of the patient's needs and to provide the patient with an opportunity to ask questions. The clinic interaction will enhance the doctor-patient relationship and promote optimal quality patient care. The clinic consultation should ideally be conducted face-to-face in a private room, accompanied by a radiology nurse where applicable. The practice of a virtual clinic is now well recognised (3), and this can be easily adapted to IR practice. The clinic discussion should evaluate the medical history, the scope of the symptoms, identify risk factors (e.g. allergies, bleeding disorders, anticoagulation, renal impairment), and allow a physical examination where applicable. The logistics of the proposed treatment and the consent process are best discussed during the clinic consultation.

Interventional radiologists must secure allocated time in their job plans to see patients in clinics negotiation with their Clinical Directors and trust management. In the same way that surgeons see patients referred to them in clinics, interventional radiologists are no different, and must have allotted time and clinic rooms to assess patients referred for IR procedures. The relevant society documents can be used to support these discussions if necessary (1, 4, 5).

Finally, similar to other clinics in hospitals, IR clinics are revenue producing, and increase the income for interventional radiology and the hospital trust. This fact should be stressed by IRs to their managers when negotiating time in their job plans and for facilities to hold the clinics.

### The Consent Process

This involves an explanation of the procedure and a discussion of the benefits and possible adverse events to the patient (or their parent or legal representative). The language used should be simple and clear. Alternative therapeutic options should also be discussed. Patients should have the opportunity to ask questions and sufficient time should be allocated between obtaining informed consent and the procedure unless in the emergency situation. Ideally, the patient should receive an information leaflet pertaining to the procedure and the consent form should be adequately documented and uploaded online into the patient’s records. The BSIR and CIRSE produce several patient information leaflets that can be used to assist the consent process.

### Follow-up Assessment

Post-procedure follow-up involves the immediate aftercare and subsequent post-discharge review. Post-procedure ward rounds are essential for informing patients of the immediate outcome, ensuring satisfactory aftercare, and fostering a good doctor-patient relationship. The practice of post-discharge follow-up that involves imaging assessments and clinic reviews may vary subject to local practice. However, interventional radiologists are encouraged to lead the follow-up programme or at least manage it in conjunction with the clinical teams. It is also important to ensure good communication with the primary care providers, ideally by means of discharge letters.

### Job Plan to Include Clinical Practice Commitments

It is essential that all IR clinical practice commitments are incorporated into an interventional radiologist’s job plan and that appropriate programme activities are recognised and allocated. Clinical activities such as pre-procedure assessment clinics, post-procedure reviews, and time taken for documentation and clinical correspondence, should all be included in the job plan. The practice of IR is changing. Appropriate resources and support should be provided as recommended by the RCR job planning guide (4, 5).

**References**

8.1: CIRSE Clinical Practice Manual. Mahnken AH, Boullosa Seoane E, Cannavale A, de Haan MW, Dezman R, Kloeckner R, O'Sullivan G, Ryan A, Tsoumakidou G. Cardiovasc Intervent Radiol. 2021 Sep;44(9):1323–1353.
8.2: Joint British Society of Interventional and British Society of Haematology on Interventional Radiology procedure bleeding risk guidance. https://www.bsir.org/media/resources/IR_BLEEDING_RISK_GUIDANCE_FINAL_BSIR_BSH_2022.pdf
8.3: Virtual clinics in Highly Specialised Services (HSS): guidance for services supporting patients with rare and complex and multi-system disorders. NHS England. 7 March 2023. https://www.england.nhs.uk/long-read/virtual-clinics-in-highly-specialised-services-hss-guidance-for-services-supporting-patients-with-rare-and-complex-and-multi-system-disorders/
8.4: Clinical radiology job planning guidance for consultant and SAS doctors 2022. Royal College of Radiologists 2022. https://www.rcr.ac.uk/system/files/publication/field_publication_files/cr-job-planning-guidance-2022.pdf
8.5: Job planning for interventional radiology an addendum to Guidance for job planning in clinical radiology. Royal College of Radiologists 2018 https://www.rcr.ac.uk/sites/default/files/bfcr139a_ir_job_planning_addendum.pdf

## TRAINING

The RCR census data 2021 (1) showed that 50% of trust/health boards had inadequate IR service provision. This was mainly due to an IR workforce shortfall of 28%. This shortfall was based on the requirement of six consultants needed to provide a viable 24/7 service at each trust. The positive trend that was noticed in the census was a 4% annual growth in the IR workforce from 2016 to 2021. However, the average annual growth of vascular IRs has only been 2% in comparison to non-vascular IRs at 8% and interventional neuroradiologists at 7%.

The new 2023 workforce census has demonstrated a growth of IR consultants by 2%, which has been mostly vascular with no growth in non-vascular or neuro IRs. The number of consultants leaving the service has increased (in 2022, 39 WTE consultants left the IR workforce, compared with 18 consultants in 2021) with the mean age of the consultants leaving the workforce reducing over a 5-year period (55 years in 2018 to 44 years in 2022) (2).

The BSIR along with the RCR have made a concentrated effort to address this problem. With 33% more doctors commencing their specialty IR training in 2021 in comparison to 2016, and the advent of the introduction of run-through training for IRs starting at ST1 level, a part of the workforce shortage is being addressed (3). An acceptance of the strategy of offering ST1 IR training posts by radiology training schemes and deaneries across the UK is the key for success. The BSIR is actively working with all stakeholders to advance this cause.

**Fig. 1:**
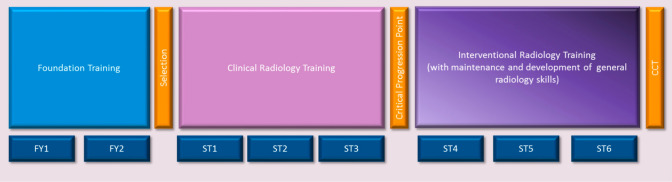
Training pathway for interventional radiology

As stated above, run-through training in IR at the ST1 level has just been introduced. Until it becomes more widely accepted by deaneries and radiology training leads, the standard route for IR training will continue to be the entry from ST3 to subspecialty training year ST4 for radiology trainees who have completed 3 years of clinical radiology curriculum requirements. Post-ST3 subspecialty training runs for another 3 years with competencies to be achieved in the IR curriculum before the completion of training (CCT) is offered in IR (4) (Fig. 1).

Since 2022, entrants to radiology training have been able to state their preference to start IR training from the ST1 level. These Clinical Radiology (Intervention) (CR(I) ST1 trainees have exposure to IR each week for the first three years of training and then automatically progress to subspecialty training for years ST4 to ST6 once they have competed the FRCR examinations.

Therefore, the CR(I) trainees have a continuous training commitment to IR from the first year, unlike the previous pathway. The aspiration is that this will help to recruit more trainees committed to IR at the start of their radiology career (5). The introduction of this new ST1 IR entry along with the traditional ST4 entry option provides a choice of options for radiology trainees who want to consider IR as a career.

Some foundation training schemes in the UK have started to offer foundation year positions in radiology. There is a need for IRs and radiologists in general to work with medical schools to increase awareness of IR among medical students and foundation year doctors to increase uptake into the subspecialty.

Finally, it is essential that the new ST1 IR training route is adopted widely by deaneries and radiology training leads to ensure that this new initiative is a success and does not fail similar to previous attempts to improve IR training, such as the now discontinued Focussed Individual Training in IR (FIT). It is incumbent on all radiologists, whether they are IR or not, to promote IR training commencing at the ST1 level to meet the workforce challenge of the ever-increasing demand for IR services.

**References**

9.1: https://www.rcr.ac.uk/clinical-radiology/rcr-clinical-radiology-census-report-2021/detailed-census-data-clinical-radiology
9.2: https://www.rcr.ac.uk/sites/default/files/documents/rcr_clinical_radiology_workforce_census_2023.pdf
9.3: Uberoi R, Haslam P, Suresh P, Morgan R. Meeting the needs of future UKinterventional radiology: launch of year 1 interventional radiology trainee program. Clin Rad 2023. 10.1016/j.crad.2023.03.009
9.4: https://www.rcr.ac.uk/clinical-radiology/specialty-training/curriculum/interventional-radiology-curriculum
9.5: https://medical.hee.nhs.uk/medical-training-recruitment/medical-specialty-training/clinical-radiology/core-clinical-radiology/overview-of-core-training/applying-for-st1-training

## ACCREDITATION FOR IRs

### Why Accreditation is Necessary

The scope of interventional radiology has rapidly expanded over recent years in both elective and emergency care. It is essential to ensure that newly qualified IRs have the necessary skills to provide best practice patient care as well as the ability and resources to keep up with the expected future fast-paced development of IR. In the UK, IR services are provided according to a wide variety of models.

Formal accreditation for our specialty is long overdue. Professional accreditation refers to endorsement by a Professional, Statutory, or Regulatory Body (PSRB) based on meeting national benchmarks (1). Development of a robust system where it is possible to confirm the professional competence of an IR would benefit the subspecialty of IR and would improve patient safety.

### Accreditation Options for IR in the UK

#### Why FRCR is Not Adequate

The most recent RCR interventional radiology curriculum from August 2020 (2) defines the knowledge and competencies required for IR trainees. The curriculum aims to develop specialists with good general radiology skills, practical experience of minimally invasive procedures and the ability to clinically manage patients undergoing IR procedures. The curriculum provides a training framework, describing the standard required to achieve the subspecialty Certificate of Completion of Training (CCT) and the expected levels of progress throughout training. Trainees are able to further subspecialise in vascular and/or non-vascular interventional radiology or in interventional neuroradiology.

The FRCR examination offers an objective evaluation of a trainee’s knowledge and competence in general diagnostic radiology which, alongside workplace-based assessments, provides evidence towards a Diagnostic Radiology CCT. However, the knowledge required to practice IR extends significantly beyond that required for FRCR. There is no objective assessment of IR specific knowledge for a trainee, nor for a Certificate of Eligibility of Specialist Registration (CESR) applicant, prior to issue of a CCT in Radiology with a subspecialty of Interventional Radiology.

#### European Board of Interventional Radiology

The European Board of Interventional Radiology (EBIR) operated by the Cardiovascular and Interventional Radiology Society of Europe (CIRSE) is a voluntary supplemental examination designed to evaluate interventional radiologists on the clinical and technical knowledge necessary to carry out safe and effective treatments for patients. EBIR certification does not replace any national training or licensing, but does provide high quality evidence of an individual’s knowledge base.

#### Alternative Options in the UK

One option would be to continue with training as at present but to make the EBIR mandatory for all applicants for CCT with subspecialty in IR. This would require a Memorandum of Understanding with CIRSE to use the EBIR examination under licence. It would likely be a challenge to secure agreement from the RCR to do this. Practically speaking, it may be easier to achieve this if IR was a separate faculty within the RCR.

The development of a UK-based exit examination modelled on the existing exit examinations provided by the Royal Colleges of Surgeons (3) is an alternative. However, this would be time consuming and would significantly overlap with the EBIR.

Finally, the development of a UK practical examination (technical competency) for interventional radiologists to complement the FRCR and EBIR could be considered. This would complement the EBIR, perhaps with patient-based scenarios and simulator procedures. Although time consuming and expensive to develop and run, this could bring added value to existing options.

In reality, the most practical and least expensive option would be to mandate that all IR trainees and CESR applicants take and pass the EBIR before taking up a consultant appointment in interventional radiology.

**References**

10.1: https://arcs.qmul.ac.uk/quality-assurance/professional-accreditation/#:~:text=A%20professionally%20accredited%20programme%20is,graduates%20meets%20national%20professional%20benchmarks
10.2: https://www.rcr.ac.uk/sites/default/files/interventional_radiology_curriculum_2020.pdf
10.3: https://www.jcie.org.uk/content/content.aspx?ID=12

## ACCREDITATION FOR IR CENTRES

All centres providing interventional radiology services should aspire to perform to a recognised level of care.

BSIR has operated an exemplar site process since 2016. CIRSE administers certification to centres providing interventional oncology procedures via the International Accreditation System for Interventional Oncological Services (IASIOS). Both processes demonstrate adherence to best practice in multiple domains, allowing commissioners and end users a benchmark assurance of high-quality IR or oncological IR care.

### BSIR Exemplar Status

Exemplar status via the BSIR can be Pilot level or Full level (1). At inception, the project was supported by the BSIR, the RCR, the National Imaging Board and the Department of Health. The intention was to improve quality and access to interventional services across the UK. Although not providing formal accreditation, BSIR Exemplar status does demonstrate an ongoing commitment by individual IR departments to improving quality and access to IR. Judged against four key domains: scope of services, providing good quality care, patient focus and service improvement. Pilot sites show substantial but incomplete compliance, with local clinical leadership commitment to work towards Full compliance/status.

## IASIOS

A fee paying formal accreditation offering certification to any centre offering interventional oncology services. Sites must perform over 150 annual IO procedures and can achieve three levels of certification benchmarked against a comprehensive set of criteria (2).

**References**

11.1: https://www.bsir.org/safety-quality/bsir-quality-improvement-initiative/. Accessed 14/03/23.
11.2: https://www.iasios.org/. Accessed 14/03/23.

## GOVERNANCE

Interventional radiology, like any other clinical specialty, is required by clinical governance to ensure that the quality of work is regularly assessed and improved. There are several agreed quality standards in interventional radiology that are set by national and international bodies (e.g. NICE, RCR, BSIR and CIRSE), which IR units should follow when assessing their own practice and outcomes.

Interventional radiology units should have an active audit programme and should hold regular morbidity and mortality meetings where complications can be discussed in a safe environment, adopting a no-blame culture, and encouraging the sharing of experience and the dissemination of learning. Examples of good practice and innovative ways of managing a complex case can also be discussed with the aim of ultimately improving patient care and outcomes.

National registries provide a consistent way to measure the quality and outcomes of patient care, supporting developments in service provision, and can provide comparative figures on performance between individuals and centres. Contributing to registries allows hospitals to ascertain what they are doing well, and can highlight areas that require improvement. Examples of registries pertaining to IR include the National Vascular Registry, which collects data on two IR index procedures (lower limb angioplasty and endovascular abdominal aortic aneurysm repair). Data submission to national registries forms a vital part of IR practice, and it can play an important role in supporting the accreditation of IR departments. Data submission to national registries should be mandated and be part of an interventional radiologist’s job plan. This also allows assessment of an individual’s performance, which is essential for appraisal and revalidation.

The BSIR quality improvement programme offers IR units the opportunity to self-assess against set criteria with the ultimate objective of being awarded exemplary status. All IR units are encouraged to participate in this quality improvement initiative, also known as the Exemplar programme (see above).

The BSIR has been involved in discussions with various funding bodies including the RCR to set up a national registry of IR procedures. This would involve the submission of every IR procedure by all interventional radiologists as they are performed and wherever they are performed. This would have several benefits, including a national knowledge of the overall numbers of IR procedures performed on an annual basis in the UK; knowledge about individual outcomes that could be used for appraisal and governance; knowledge of the gaps in service provision for certain specialist IR procedures, and not least would create an important tool for research. Although there is significant desire on the part of the BSIR to set up such an IR registry, the main barrier to date has been a lack of funding. Nevertheless, a UK IR Registry remains an ongoing project for the BSIR to continue to work towards.

**References**

12.1.: National Vascular Registry (https://www.vsqip.org.uk)
12.2.: British Society of Interventional Radiology (https://www.bsir.org/safety-quality/bsir-quality-improvement-initiative)

## MAJOR CLINICAL INDICATIONS FOR INTERVENTIONAL RADIOLOGY

### Management of Haemorrhage

Control of haemorrhage is one of the cornerstones of emergency interventional radiology. With ongoing technical improvements in catheter/microcatheter equipment and embolic agents such as coils, vascular plugs and liquids, the means to perform emergency embolisation procedures has never been more available. As the success of embolisation procedures for haemorrhage increases, the demands for the IR service increase both in and out of hours.

***Gastrointestinal haemorrhage—***In upper gastrointestinal (GI) haemorrhage, IR embolisation plays a key role in treating patients who fail medical and endoscopic intervention. For patients with refractory oesophageal or gastric variceal bleeding, IRs may offer life-saving therapy by performing a transjugular intrahepatic portosystemic shunt (TIPSS). For lower GI haemorrhage, recent guidance from the British Society of Gastroenterology has favoured the use of IR embolisation over surgical intervention in unstable patients with evidence of active bleeding on CT Angiography (1).

***Traumatic haemorrhage—***The development of major trauma centres (MTCs) for the severely injured trauma patient has led to centralisation of services. NHS guidance indicates that MTCs should have timely access to an interventional radiology consultant who can treat traumatic haemorrhage due to visceral injury (spleen/kidney/liver/etc.) and pelvic trauma by embolisation. Interventional radiologists are also important for the treatment of peripheral vascular injury by embolisation and stent-grafting, and the management of aortic injury by abdominal or thoracic aortic endografts.

***Haemoptysis—***Bronchial artery embolisation is an important interventional radiological treatment for patients with massive haemoptysis. Recent guidelines produced by CIRSE indicate that following multi-detector CT to identify anatomy, bronchial artery embolisation can be performed by interventional radiologists with sufficient expertise with high technical success rates of > 90% (2).

***Obstetric haemorrhage—***Obstetric haemorrhage remains one of the major causes of maternal death after delivery. Interventional radiologists are essential in any hospital with a maternity unit as they can perform rapid and emergent embolisation of pelvic arterial branches for post-partum haemorrhage and save maternal lives. Interventional radiologists also play a key role in the management of patients with placental anomalies. For these patients who have been identified as being at high risk prior to delivery, obstetricians may decide in consultation with their IR colleagues for the IRs to place compliant occlusion balloons in both internal iliac arteries before delivery. These can be inflated where required during and immediately following delivery to minimise haemorrhage. If haemorrhage occurs post-balloon deflation, emergent uterine artery embolisation can be performed by the interventional radiologist.

### Peripheral Vascular Disease

One of the fundamental areas of treatment for interventional radiologists is peripheral vascular disease (PVD), which is one of the most common health issues affecting the UK population. It is estimated that PVD affects approximately 20% of people over the age of 60 (3). Interventional radiology methods for treating peripheral arterial occlusive disease include angioplasty and/or stenting using plain or drug-eluting/coated devices as well as more advanced methods such as atherectomy and intravascular lithotripsy. Compared to surgery, endovascular treatment is cost-effective, often serves as a first line method of treatment, and provides similar results to surgery in patients who lack a suitable venous conduit for bypass (4).

Interventional radiologists also play a key role in the management of patients with acute limb ischaemia using intra-arterial transcatheter thrombolysis and/or percutaneous thrombectomy. These procedures utilise a variety of dedicated devices from straightforward aspiration thrombectomy to mechanical thrombectomy.

### Aortic Disease

Interventional radiologists play an important role in the management of aortic aneurysms, aortic dissection, and aortic trauma. This may involve the insertion of standard or complex aortic endografts, often as a team in collaboration with vascular surgical colleagues. Interventional radiologists also play a key role in the management of complications of aortic endografting such as the embolisation of endoleaks.

### Uterine Fibroids

Uterine fibroids are the most common type of tumour in women of reproductive age (5). The quality of life of approximately half of these women is adversely affected by significant symptoms (6). These women can be treated with uterine artery embolisation (UAE) by interventional radiologists. Uterine artery embolisation provides a much less invasive alternative to surgery and is often the only option for women who wish to preserve their uterus. There is no significant difference in long-term results between UAE and surgical treatment. Therefore, every female patient who requires treatment for uterine fibroids should be offered UAE if appropriate (7).

### Venous Thromboembolic Disease

Interventional radiologists are essential in the management of patients with venous disease. One of the key roles of IRs in venous disease is in the management of acute and chronic venous occlusive disease.

Venous thromboembolic disease poses a major impact to the healthcare economy not just in terms of mortality but also morbidity. The development of a post-thrombotic limb after ilio-femoral deep vein thrombosis may lead to major and lifelong consequences of chronic venous insufficiency for a group of patients who are often young. More severe cases may present acutely with a threatened limb which requires prompt treatment (8).

Catheter directed thrombolysis may benefit patients with acute ilio-femoral deep vein thrombosis. More recent advances in mechanical thrombectomy technologies have led to an increase in efficiency of thrombus clearance (9). The evidence indicates that young patients with ilio-femoral deep vein thrombosis and low bleeding risk would benefit from thrombus clearance—a position that has been adopted in many national guidelines around the world.

Deep venous stenting is helpful in certain specific situations, both in the acute and the chronic situation. The advent of dedicated venous stent technologies has improved outcomes. Interventional radiologists may use intravascular ultrasound to assist with stent sizing and deployment.

Inferior vena cava (IVC) filters are indicated in specific circumstances to reduce the risk of pulmonary emboli—e.g. when a patient with a deep vein thrombosis cannot be treated by anticoagulant therapy. Once deployed, scheduled removal of the filter should be undertaken at an agreed time interval at a later date. The evidence for pulmonary embolism reduction after IVC filter insertion has been well established for more than 20 years. Recent trends in management have focussed on correct patient selection for filter placement, protocols for removal and adequate follow up arrangements.

The emerging field of pulmonary embolism thrombectomy / catheter directed thrombolysis has led to some potentially promising results but as yet remains limited to certain centres.

### Vascular Access

Interventional radiologists perform various vascular access procedures, especially for long-term venous access in both adult and paediatric patients. The most common venous access procedures are peripherally inserted central catheters, tunnelled central venous catheters and tunneled central venous ports. Potential uses of these devices include haemodialysis, chemotherapy, plasmapheresis, parenteral nutrition and long-term antibiotic treatment. With cancer therapy identified as one of the clinical priorities in the NHS long-term plan and a general increase in difficult vascular access patients, interventional radiology services to provide venous access are imperative to assist in improving patient outcomes.

### Dialysis Access Insertion and Maintenance

As of 2020, there were approximately 25,532 adults and 100 children on long term haemodialysis, and more patients are being added every year (10). The scope of interventional radiology includes performing access procedures for dialysis, such as placing tunneled and non-tunneled dialysis lines, creating percutaneous endovascular arteriovenous fistulas, and maintaining surgically created fistulas.

Percutaneous fistuloplasty and stent insertions, and percutaneous thrombectomy procedures are just some of the interventional radiology techniques that are performed to maintain the patency of a malfunctioning dialysis fistula.

### Urological Obstruction

An obstructed kidney is a core urgent referral to all IR departments, with an infected obstructed kidney being a medical emergency. Although there is selective evidence that the routes of decompression, i.e. urological retrograde stenting versus radiological percutaneous nephrostomy (PCN) are equivalent (11), a PCN is quicker and has a higher success rate (12), especially with distal obstruction or significant ureteral stone disease. The facility to perform PCN must be available at all times (24/7) to all acute hospitals either onsite or at a neighbouring hospital through a prearranged formal agreement via a network arrangement.

Unless severe urosepsis is present at the time of nephrostomy, a ureteric stent should be inserted at the same time as drainage of the pelvicalyceal system.

Percutaneous nephrostomy is also required in non-obstructed scenarios when trying to divert urine away from the renal pelvis if there is a renal or ureteric leak or a ureteric fistula.

### Biliary Obstruction

Percutaneous biliary drainage and stenting may provide prompt drainage of an obstructed biliary tract. Relief of bile duct obstruction is required urgently if there is a suspicion of biliary sepsis in the presence of bile duct obstruction.

Although percutaneous biliary drainage (PBD) with a metallic or plastic stent has a higher technical success rate, endoscopic retrograde cholangiopancreatography (ERCP) has become the first line management of biliary obstruction outside of the liver transplant population (13). As a result, the number of PBDs performed has fallen over the last two or three decades and is today reserved for failed ERCP or for complications after liver transplantation. The provision of PBD should ideally only be performed by high volume upper GI centres or hepatobiliary centres with prearranged referral agreements from neighbouring units. The requirement of emergency out of hours PTC is rare.

### Nutrition and Feeding Tubes

Patients who are not able to eat or drink either because of neurological disorders or obstructing lesions of the oesophagus can receive nutrition via a variety of feeding tubes that can be inserted by interventional radiologists.

These vary from per-oral or per-nasal tubes, e.g. nasogastric and nasojejunal tubes to percutaneous gastrostomy and gastrojejunostomy tubes. Interventional radiologists play a major role in assisting nutrition for many patients with severe disease that prevents them from eating and drinking via the normal route.

### Cancer Treatment: Interventional Oncology

Interventional radiologists have developed many techniques to treat and palliate cancer. As a result, IRs have created the specific entity of interventional oncology. Interventional oncology is increasingly regarded as the 4th pillar of cancer care alongside surgery, radiotherapy and chemotherapy. The main interventional oncology techniques are percutaneous tumour ablation and transcatheter tumour embolisation.

***Tumour ablation—***Since the first case reports of chemical ablation of hepatocellular carcinoma (14) and radiofrequency ablation of renal cell carcinoma (15) were published as far back as 1993, image-guided percutaneous ablation of malignant tumours has become an established, effective treatment especially in the liver, kidney, and other organs. The indication, method and choice of cytotoxic energy deposition continues to develop. The main methods of ablation are radiofrequency ablation, microwave ablation, irreversible electroporation and cryotherapy. The choice of ablation treatment is multifactorial including anatomy, comorbidity and patient and physician preference.

***Tumour embolisation—***Interventional radiologists play a major role in the treatment of many cancers by transarterial embolisation. The liver is the organ that is most frequently targeted for transarterial embolisation. Either primary liver tumours (hepatocellular carcinoma—HCC) or secondary liver tumours (e.g. colorectal metastases) can be treated by embolisation with or without a chemotherapeutic drug (chemoembolisation). The dual blood supply of the liver makes it an ideal target for intra-arterial embolotherapy. Standardisation of embolisation techniques and chemotherapeutic agents, and ongoing technical improvements have led to improved outcomes in patients with primary liver cancer.

Bland embolisation of hepatocellular carcinoma with particles or microspheres is still performed, although increasingly units have moved towards mixing the embolic agent with a chemotherapeutic drug such as Doxorubicin (transarterial chemoembolisation—TACE). Transarterial (chemo)embolisation is less commonly performed for secondary liver tumours although it remains an option in certain selected cases, e.g. symptomatic neuroendocrine tumour metastases.

More recently, NICE guidance has approved the use of Selective Internal Radioembolisation therapies (SIRT), typically using microspheres bound with Yttrium-90, which are injected into the arteries supplying the tumour. This treatment is available for patients with colorectal liver metastases as well as those with HCC. MDT discussion is required as well as close collaboration with nuclear medicine colleagues.

For intermediate stage HCC, survival with TACE could be expected to be greater than 2.5 years. The success of this therapy and subsequently SIRT has led to a defined clear role for these treatments within most national and international guidelines (16).

Embolisation outside the liver is less often performed for oncological control, but may be helpful in specific symptomatic situations (e.g. relief of haematuria in renal cell carcinoma -RCC) or as an adjunct to other treatments, e.g. RCC bone metastasis embolisation to reduce haemorrhage during subsequent orthopaedic fixation.

***Palliation of advanced cancer—***Interventional radiologists play a crucial role in improving the quality of life of patients with incurable malignancy. This can range from simple procedures (e.g. rapid transfer of patient to and from a hospice for paracentesis during end-of-life care) to more complex procedures (e.g. thermal ablation of painful skeletal metastatic disease). Each cancer unit IR department may not offer the full spectrum of interventions, but must have arrangements in place with neighbouring hospitals if there is a clinical need that they cannot meet. This can be facilitated via the regional Cancer Alliance.

The decision to not intervene in a palliative setting is an important choice for patients when quality of life is the primary outcome.

### Stroke

Approximately 20% of stroke survivors die within the first year and over 50% are left with long term disability. This costs the NHS and the wider economy approximately £7billion per year (17). The highest degree of disability occurs in patients with a proximal large artery occlusion (40% of strokes), who may not respond well to thrombolytic agents. Approximately 10–12% of these patients are suitable for mechanical thrombectomy. Mechanical thrombectomy for proximal intracerebral artery occlusion can prevent irreversible cerebral ischaemia and prevent or limit long term disability (18). Mechanical thrombectomy is possibly one of the most significant interventions to be developed since acute coronary intervention.

Stroke thrombectomy is undertaken using stent-retrievers, sometimes with supplemental thrombus aspiration. This technique has been shown to produce the best outcomes (19).

The procedure is performed by clinicians trained in vascular minimally invasive procedures with specific training in mechanical stroke thrombectomy. In the UK this is predominantly interventional neuroradiologists and interventional radiologists.

The GMC have provided specifications for a credential in stroke thrombectomy, which clinicians from other specialties will have to complete in order to undertake this procedure.
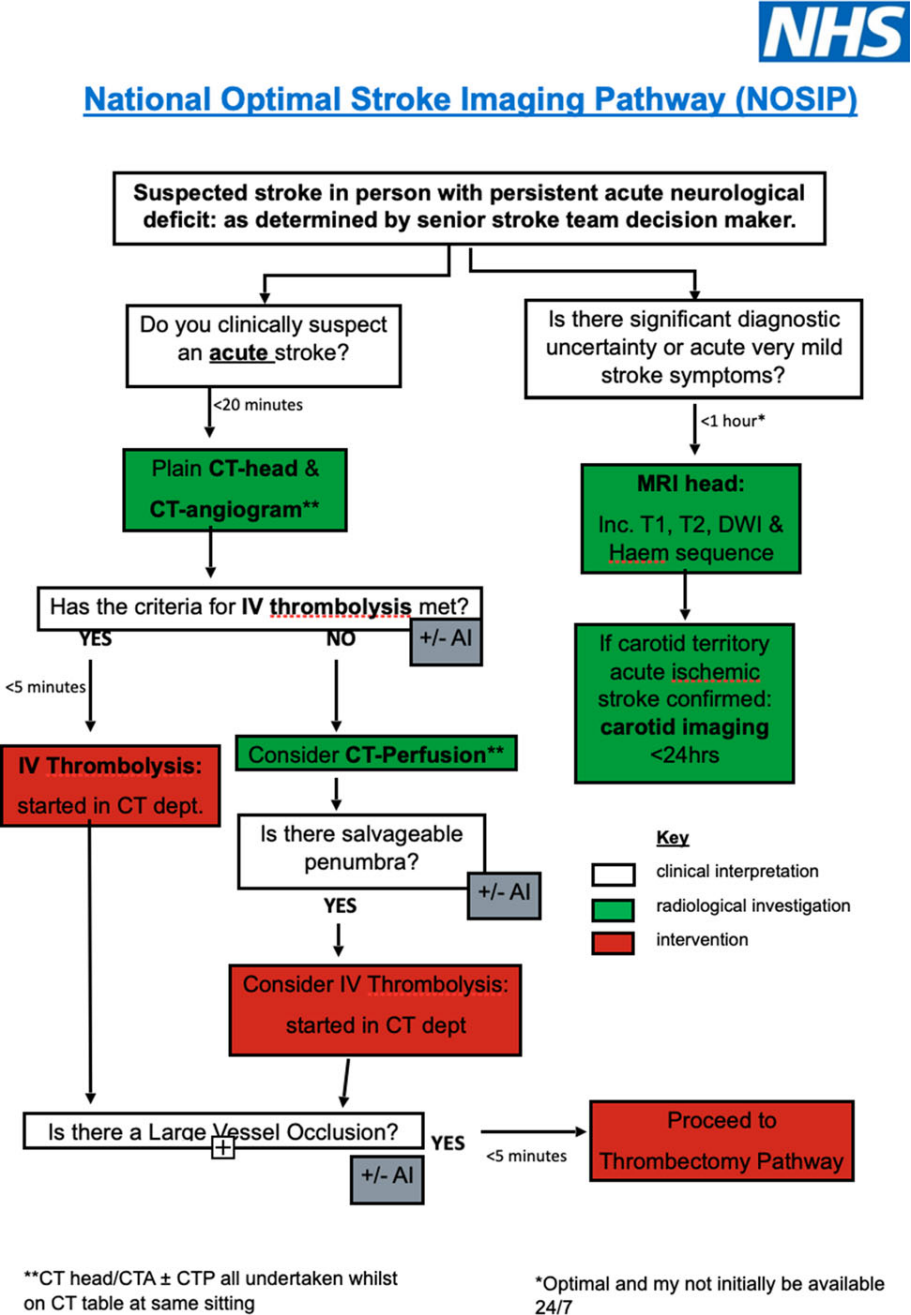


The DAWN and DEFUSE-3 (20, 21) studies revealed that mechanical thrombectomy was more effective than standard care alone for specific individuals with large vessel ischaemic stroke, who presented with symptoms within 24 h of onset. As a result, the current guidelines have expanded the window for thrombectomy to 24 h after the onset of symptoms.

An efficient stroke thrombectomy service requires clinicians who are trained in stroke thrombectomy. The service should ideally be funded and staffed to provide a 24-h service. The procedure should be performed in a centre with dedicated neuroradiological imaging and stroke physicians, to enable the correct patients to be selected for treatment.

**References**

13.1.: Diagnosis and management of acute lower gastrointestinal bleeding: guidelines from the British Society of Gastroenterology. Oakland K, et al. Gut 2019;0:1–14. 10.1136/gutjnl-2018-317807
13.2.: CIRSE Standards of Practice on Bronchial Artery Embolisation. Kettenbach et al. Cardiovasc Intervent Radiol. 2022; 45(6): 721–732.
13.3.: Prevalence | Background information | Peripheral arterial disease | CKS | NICE [Internet]. [cited 2023 Mar 26]. Available from: https://cks.nice.org.uk/topics/peripheral-arterial-disease/background-information/prevalence/
13.4.: Farber A, Menard MT, Conte MS, Kaufman JA, Powell RJ, Choudhry NK, et al. Surgery or Endovascular Therapy for Chronic Limb-Threatening Ischemia. N Engl J Med. 2022 Dec 22;387(25):2305–16.
13.5.: Stewart E, Cookson C, Gandolfo R, Schulze-Rath R. Epidemiology of uterine fibroids: a systematic review. BJOG Int J Obstet Gynaecol. 2017;124(10):1501–12.
13.6.: Borah BJ, Nicholson WK, Bradley L, Stewart EA. The Impact of Uterine Leiomyomas: A National Survey of Affected Women. Am J Obstet Gynecol. 2013 Oct;209(4):319.e1-319.e20.
13.7.: Daniels J, Middleton LJ, Cheed V, McKinnon W, Sirkeci F, Manyonda I, et al. Uterine artery embolization or myomectomy for women with uterine fibroids: Four-year follow-up of a randomised controlled trial. Eur J Obstet Gynecol Reprod Biol X. 2021 Nov 20;13:100139.
13.8.: NICE guideline NG158 - Venous thromboembolic diseases: diagnosis, management and thrombophilia testing
13.9.: European Society for Vascular Surgery (ESVS) 2022 Clinical Practice Guidelines on the Management of Chronic Venous Disease of the Lower Limbs – De Maessneer et al. EJVS 63;2: 184–267
13.10.: 24th Annual Report - data to 31/12/2020 | The UK Kidney Association [Internet]. [cited 26]. Available from: https://ukkidney.org/audit-research/annual-report/24th-annual-report-data-31122020
13.11.: Pearle MS, Pierce HL, Miller GL, Summa JA, Mutz JM, Petty BA, Roehrborn CG, Kryger JV, Nakada SY. Optimal method of urgent decompression of the collecting system for obstruction and infection due to ureteral calculi. J Urol. 1998 Oct;160(4):1260–1264. PMID: 9751331.
13.12.: Mokhmalji H, Braun PM, Martinez Portillo FJ, Siegsmund M, Alken P, Köhrmann KU. Percutaneous nephrostomy versus ureteral stents for diversion of hydronephrosis caused by stones: a prospective, randomized clinical trial. J Urol. 2001 Apr;165(4):1088–92. PMID: 11257644.
13.13.: Van Eecke E, Degroote H, Vanlander A, Hindryckx P. Outcome of primary ERCP versus primary PTC for biliary drainage in malignant hilar biliary strictures: a systematic review and meta-analysis. Surg Endosc. 2022 Oct;36(10):7160–7170. 10.1007/s00464-022-09413-5. Epub 2022 Aug 8. PMID: 35941311.
13.14.: Castells A, Bruix J, Bru C, Fuster J, Vilana R, Navasa M, Ayuso C, Boix L, Visa J, Rodés J. Treatment of small hepatocellular carcinoma in cirrhotic patients: a cohort study comparing surgical resection and percutaneous ethanol injection. Hepatology. 1993 Nov;18(5):1121–1126. PMID: 8225217.
13.15.: Gervais DA, McGovern FJ, Wood BJ, Goldberg SN, McDougal WS, Mueller PR. Radio-frequency ablation of renal cell carcinoma: early clinical experience. Radiology. 2000 Dec;217(3):665–672. 10.1148/radiology.217.3.r00dc39665. PMID: 11110926.
13.16.: BCLC strategy for prognosis prediction and treatment recommendation: The 2022 update. Reig et al Journal of Hepatology 2022 vol. 76 j 681–693
13.17.: NHS England Evidence review: Mechanical thrombectomy for acute ischaemic stroke in the anterior cerebral circulation (2023 draft)
13.18.: Berkhemer OA, Fransen PS, Beumer D,et al; MR CLEAN Investigators. A randomized trial of intraarterial treatment for acute ischemic stroke. N Engl J Med. 2015 Jan 1;372(1):11–20. 10.1056/NEJMoa1411587.
13.19.: Nogueira RG, Lutsep HL, Gupta R, et al; 2012. Results from the TREVO 2 Study (Thrombectomy REvascularization of large Vessel Occlusions in acute ischemic stroke): Randomized data comparing Trevo with Merci for thrombectomy in acute stroke. *Cerebrovascular Diseases,* 33**,** 57.
13.20.: Nogueira RG, Jadhav AP, Haussen DC, et al; DAWN Trial Investigators. Thrombectomy 6 to 24 Hours after Stroke with a Mismatch between Deficit and Infarct. N Engl J Med. 2018 Jan 4;378(1):11–21. 10.1056/NEJMoa1706442. Epub 2017 Nov 11. PMID: 29129157.
13.21.: Albers GW, Marks MP, Kemp S, et al; DEFUSE 3 Investigators. Thrombectomy for Stroke at 6 to 16 Hours with Selection by Perfusion Imaging. N Engl J Med. 2018 Feb 13.22;378(8):708–718. 10.1056/NEJMoa1713973. Epub 2018 Jan 24. PMID: 29364767; PMCID: PMC6590673.

## PAEDIATRIC INTERVENTIONAL RADIOLOGY

Interventional radiology plays a key role in healthcare provision for children, just as it does for adults. As with adult care, paediatric IR (PIR) provides adjunct services such as biopsies, vascular access and feeding tube insertions as part of wider bundles of care but also delivers standalone IR procedures for children such as embolisation, sclerotherapy, TIPS, thrombectomy and interventional oncology care. The benefits of IR are just as relevant for children, their families, and the NHS, as they are for adult care: quicker recovery times, shorter hospital stays, no surgical scars, lower risk of surgical sequalae, lower risk interventions and a greater likelihood of preserving vital structures in patients often with 60 more years of life ahead of them (1–3).

PIR provision has been slow to develop in the UK for a number of reasons. These include a severe shortage of both training and consultant posts, lack of recognition of the value of PIR by commissioners, hospitals and policymakers and an inability to generate meaningful PIR data due to coding obstacles. Expertise has matured in a small number of specialist centres but there are very large parts of the UK where children cannot access PIR services and instead are offered open surgical alternatives or no intervention at all.

Recent guidance from the RCR has clearly described the hurdles to PIR growth in the UK and has made a series of robust recommendations to improve IR services for children (4). These include:

- the creation of nationally commissioned networks of care linked to Integrated Care Systems and paediatric surgical operational delivery networks.
- a sustained growth in PIR consultant posts over the next 15 years.
- improved national capacity and flexibility in PIR training and cross skill training for other healthcare professionals such as adult interventional radiologists, paediatric surgeons, diagnostic radiologists, nurses and radiographers.
- PIR must be incorporated into service specifications and assessment standards for major trauma services.
- a significant refinement to how NHS PIR data is coded and collected.
- above all, every hospital in the UK that offers care to children must have a robust plan for local IR provision and/or the timely onward referral of patients to networked regional specialist centres.

**References**

14. 1: Mohamed H, Pastor MC, Langlois S, Cowan KN (2022) Comparing safety and adequacy between surgical biopsy versus core needle biopsy in diagnosing neuroblastoma. J Pediatr Surg 57:866–870
14.2.: Lau CSM, Chamberlain RS (2016) Ultrasound-guided central venous catheter placement increases success rates in pediatric patients. Pediatr Res 80:178–184
14.3.: Hancock-Howard R, Connolly BL, McMahon M et al (2010) Cost-effectiveness analysis of implantable venous access device insertion using interventional radiologic versus conventional operating room methods in paediatric patients with cancer. J Vasc Interv Radiol 21:677–684
14.4.: Improving paediatric interventional radiology services in the UK, The Royal College of Radiologists 2023

## WOMEN IN IR

Although there are now more women than men graduating from medical schools in the UK, women are under-represented in the interventional radiology workforce. The cause of this is multifactorial. Contributory factors include misconceptions among women regarding occupational radiation exposure and pregnancy. Many women have the impression that being an interventional radiologist is not compatible with a family life. In fact, the radiation dose levels received by IRs are routinely monitored and are usually well below the constraints that are recommended in pregnancy. Additional precautions and monitoring are also carried out during pregnancy to ensure that this is the case.

A career in interventional radiology is suitable for flexible working similar to other specialities. The ability to work part-time, or to work long days and have days off, should be negotiable and be made available to women. Interventional radiology should be seen as an interesting and satisfying career choice for women within medicine, with the option to subspecialise into a myriad of areas. As a subspecialty, it generally appeals to those who enjoy patient interaction and want to make a real difference to patients’ lives using innovative, minimally invasive procedures.

More should be done to ensure that the subspecialty is equally as attractive to women as it is to men. With significant and ongoing workforce shortages in IR, it is more important than ever to encourage women to become interventional radiologists (1–3).

**References**

15.1.: Women in interventional radiology: Insights into the subspecialty. The Royal College of Radiologists.
15.2.: Belli, AM., Englander, M. The Female Threat. *Cardiovasc Intervent Radiol* **41**, 673–674 (2018). 10.1007/s00270-018-1915-2
15.3.: Englander MJ, O'Horo SK. JOURNAL CLUB: Women in Interventional Radiology: How Are We Doing? AJR Am J Roentgenol. 2018 Oct;211(4):724–729. 10.2214/AJR.18.19938. Epub 2018 Jul 24. PMID: 30040465.

## INFORMING PATIENTS ABOUT IR PROCEDURES

This topic can be subdivided into how to inform patients about procedures for which they have been referred, and how to inform patients about interventional radiology in general.

### For Patients Referred for an IR Procedure

Patients should be provided with relevant information regarding interventional radiology procedures wherever possible. Interventional radiology departments should have access to a resource of information to give to patients who have been referred for an IR procedure. Patient information leaflets may be created in-house by IR departments or may be found online from several providers.

There is a wide selection of information available on the internet, which provide an overview of a procedure. However, this should not be used to replace outpatient consultations and discussions tailored to a patient’s specific needs. The BSIR has a good selection of procedure specific leaflets for common procedures, which departments can use and modify for their own use (1). A wider range of leaflets is available from CIRSE (2) or the Radiological Society of North America, Inc. (RSNA) at RadiologyInfo.org (3).

### Informing the Public About Interventional Radiology

One of the main challenges for interventional radiology has been to advertise to the population at large about the benefits of interventional radiology for patient care.

Patients may hear about interventional radiology from a variety of sources including from their doctor (general practitioner or hospital specialist), media reports or online resources. Engagement by interventional radiologists with primary care physicians and local specialist services helps to promote interventional radiology services and ensure that they receive up-to-date information about local IR services. Hospital trusts are encouraged to advertise the services that they provide on their websites, but can also provide information to the NHS.UK website (4) which can be searched by patients.

Despite this, there is an ongoing need for interventional radiology as a discipline to increase patient and public awareness on the national stage. This has been a project by national and international IR societies since its inception. Prominent IR leaders have proposed to change the name “interventional radiology” to another name to potentially achieve more of a breakthrough into the public consciousness. However, although the names “pin-hole surgery” and “image guided surgery” achieved some traction, no alternative name to describe IR has achieved the durability in the public’s or media’s awareness that would be required to replace the name “interventional radiology”.

In reality, the name interventional radiology is here to stay. The project to inform the public about the benefits of interventional radiology is an important ongoing mission. Interventional radiologists wherever they work must continue to highlight who we are, what we do and how we help patients.

**References**

16.1.: Patient Information Leaflets | BSIR [Internet]. [cited 2023 Mar 22]. Available from: https://www.bsir.org/patients/patient-information-leaflets/
16.2.: Patient information leaflets and posters [Internet]. CIRSE. [cited 2023 Mar 23]. Available from: https://www.cirse.org/patients/patient-infomation-leaflets/
16.3.: Radiology (ACR) RS of NA (RSNA) and AC of. Home [Internet]. Radiologyinfo.org. [cited 2023 Mar 22]. Available from: https://www.radiologyinfo.org/en
16.4.: The NHS website [Internet]. nhs.uk. 2018 [cited 2023 Mar 22]. Available from: https://www.nhs.uk/

## TURF ISSUES

A turf issue is defined as a dispute between groups over a territory, activity or a particular sphere of influence.

After the initial development of coronary angiography and angioplasty by interventional radiologists in the 1960s, interventional cardiologists dominated the development and promotion of percutaneous coronary interventions displacing interventional radiologists from cardiac angiography laboratories. The loss of coronary angiography to interventional radiology was the first example of a turf issue faced by interventional radiologists as a result of competition from another clinical discipline.

In the last two decades, other clinical specialties have been interested in performing some of the procedures that IRs have traditionally regarded as their own, e.g. peripheral vascular work and non-vascular renal interventions. In some cases, this has led to areas of conflict, sometimes serious rifts, between specialties in hospitals in terms of who should be allowed to perform specific procedures. Unfortunately, the patient is usually the main person who suffers from such “turf conflicts” wherever they occur.

It is evident that one of the main reasons why IR has lost procedures to other specialities is that unfortunately many IRs see their role as a technical one and expect to be “served” patients to treat. If IRs want to be regarded as equals by other clinicians, they must take increased responsibility for patients and adopt the tenets of Clinical Practice discussed previously.

Different subspecialities have specific skill sets and for trusts to be able to provide a full range of high quality interventional radiological and surgical services, collaboration and partnership between different specialities is essential. The ability to deliver high quality, effective and safe care requires team working and placing the patient at the centre of health care provision (1).

Procedures and operations should only be performed by medical staff who have received the requisite training to perform those procedures. No single specialty owns specific procedures. It is evident that if doctors have been adequately trained to perform an operation, they should be allowed to do so as long as the appropriate governance structures are in place to protect patient safety.

Some procedures, e.g. EVAR and complex EVAR are best performed by collaboration among specialities (e.g. interventional radiologists and vascular surgeons) to achieve the best outcomes for patients. By performing cases with other specialities, it provides the opportunity of shared learning and enhancement of operators’ skill sets for the benefit of patients. Most exemplar sites across the UK have services that have been based on successful collaborative working between different subspecialties/specialties (1).

Turf issues can be avoided by the fostering of mutual respect of the respective skills and knowledge of each discipline in the management of patients where common interests align. Recognition by trusts of interventional radiology as an important component of the hospital establishment and the service that it provides for patients is also important to promote collaboration between subspecialties/specialties for the benefits of patient care (2, 3).

Competition between clinical specialties is a fact of life and interventional radiologists should work with clinical colleagues to provide optimal care for patients wherever possible.

**References**

17.1.: 2021: Joint statement on collaboration between the Vascular Society (VS) of Great Britain & Ireland and The British Society of Interventional Radiology (BSIR).
17.2.: European Society of Radiology (ESR). Summary of the proceedings of the International Forum 2017: “Position of interventional radiology within radiology”. Insights Imaging 9, 189–197 (2018).
17.3.: R. Morgan, T. Cleveland, M. Hamady, R. Uberoi, P. Haslam, R. Kasthuri, M. Johnston, I. McCafferty. Interventional radiology in the twenty-first century: planning for the future. Clinical Radiology 76 865–869 (2021) 865e869.

## SUBSPECIALTY AND SPECIALTY STATUS

A specialty is a branch of medical practice that is focused on a defined group of patients, diseases and skills. A subspecialty offers more focus or advanced scope on a particular area within a specialty. In the UK, interventional radiology has been recognised as a subspecialty of radiology by the GMC since 2010. In the USA in 2012, the Accreditation Council for Graduate Medical Education (ACGME) acknowledged interventional radiology to be a specialty distinct from diagnostic radiology (DR) and approved the independent DR/IR residency in 2015. The Society of Interventional Radiology sets training requirements for training programmes, and the American Board of Radiology (ABR) provides a dual IR/DR certificate.

The BSIR defines an interventional radiologist as a “clinical doctor who performs image-guided procedures, fully interprets the imaging required to guide and monitor the response of those procedures, as well as providing the pre- and post-procedural care for those patients receiving imaged guided surgery procedures”. This definition implies that interventional radiologists are skilled not only in interventional radiology procedures, but also in diagnostic imaging and clinical practice, i.e. a specialist.

While imaging remains central to interventional radiology, the primary role of an interventional radiologist is the treatment of a wide spectrum of conditions rather than their diagnosis. As such, interventional radiology has diverged significantly from diagnostic radiology. Many interventional radiologists would like interventional radiology to become a specialty under the umbrella of the RCR alongside Clinical Radiology and Clinical Oncology. The benefits would include improved training from year 1, a clear definition of the IR workforce and workforce requirements, improved patient care and patient safety with IRs bearing direct responsibility for patients, access to inpatient beds, better recognition and better job satisfaction (1, 2).

An alternative model would be for interventional radiology to become a faculty within the Royal College of Radiologists. An IR faculty may provide the majority of the benefits stated above without the need to secure the permission of the General Medical Council (which would be required for IR to form a specialty).

**References**

18.1.: Morgan R, Cleveland T, Hamady M, Uberoi R, Haslam P, Kasthuri R, Johnston M, McCafferty I. Interventional radiology in the 21st century: planning for the future. Volume 76, Issue 12, December 2021, Pages 865–869
18.2.: Makris GC, Burrows V, Lyall F, Moore A, Hamady. Vascular and Interventional Radiology Training; International Perspectives and Challenges. Cardiovasc Intervent Radiol. 2021 Mar;44(3):462–472. 10.1007/s00270-020-02688-y. Epub 2020 Nov 10

## RESEARCH AND INNOVATION

Interventional radiology has been pioneering innovative treatments for the last sixty years. However, research has not progressed at the same rate as other aspects of interventional radiology or indeed clinical specialties (1). This has led to challenges in developing services, gaining the support of referring clinicians, securing funding and investment by policy makers, securing designated research time, and allocated time and space for clinics.

It is important that interventional radiologists focus on investing and engaging in high quality research for the continued progress of interventional radiology, particularly as IR faces turf issues, financial challenges and increased public scrutiny.

We summarise here some barriers experienced by interventional radiologists who wish to engage more in IR research, as well as suggesting some solutions based on the current best available evidence.

The literature reveals perceived barriers to the development of academia within the IR community (2). These challenges comprise those inherent to the subspecialty such as lack of patient ownership, variability of practice, turf issues, lack of capacity due to workforce shortage and occasionally excessive focus on technical procedures rather than holistic patient management.

There are some challenges specific to the development of IR research in the UK that are faced during various stages of the medical career journey. For example, the 2023 Radiology Speciality application process gave research a lower priority compared to other domains such as audit (3). There is a lack of dedicated IR Academic Clinical Fellowships (ACF). There is variable encouragement provided to IR trainees by trainers to engage in research, write papers and present papers at scientific meetings. IR trainees will follow the examples set by their trainers and all IRs in training roles have a responsibility to foster a research culture among their trainees. Furthermore, research is currently not a requirement for IR consultant posts. A lack of competition for consultant positions reduces the need for IR trainees to write papers, as was the case 20–30 years ago.

For consultant interventional radiologists, the main challenge to engaging in academia in interventional radiology is the general lack of time to engage in research and write papers and a lack of academic IR posts. The majority of the IR academic posts in the UK are honorary, with no designated research time or funding for supervision of MD/PHD students where most research is produced. Consequently, out of the 404 current National Institute of Health Research (NIHR) Principal Investigators, there are no interventional radiologists and very few radiologists in general (4). There is no IR-dedicated journal published in the UK, despite having several parallel societies in IR and endovascular therapies.

Nevertheless, enhanced involvement in an academic career in interventional radiology is possible and must be supported by initiatives at every level of IR. These include embarking on an Academic Foundation Programme (AFP) and an ACF as well as Research fellowships for trainees with protected and funded research time opportunities. Involvement in trainee-led research networks such as UNITE (1) and NIHR Associate Principal Investigator schemes for conducting multicentre IR projects, as well as active participation in various international IR journals can stimulate early academic interest.

In the long term, the focus of BSIR should be working towards facilitating the creation of tenured IR university positions with dedicated time for research and education. University employed academic interventional radiologists would be best placed to write and be successful at grant proposals for IR projects from the NIHR, RCR and other research societies. Academic interventional radiology is multidisciplinary in nature and IRs must work in collaboration with other members of the IR team, dedicated research nurses and clinical trials units to achieve the highest research outputs in IR.

**References**

19.1.: Mandal I, Zhong J, Borchert R, Keni S, Jenkins P, MacCormick A, Makris GC. The UNITE Collaborative: Early Experiences of Introducing Collaborative Trainee Research to Interventional Radiology in the UK. Cardiovasc Intervent Radiol. 2022 Feb;45(2):259–260. 10.1007/s00270-021-02984-1. Epub 2021 Oct 19. PMID: 34668056; PMCID: PMC8525613.
19.2.: Jenkins P, MacCormick A, Harborne K, Liu W, Mahay U, Zhong J, Haslam P. Barriers to research in interventional radiology within the UK. Clin Radiol. 2022 Dec;77(12):e821-e825. 10.1016/j.crad.2022.08.146. Epub 2022 Oct 8. PMID: 36216606.
19.3.: Jenkins P, Mandal I, Zhong J, Goh V. Research should remain a priority in 21st century radiology recruitment to training. Br J Radiol. 2023;96(1145):20221083. 10.1259/bjr.20221083
19.4.: National Institute for Health Research. Senior investigator Directory. https://si.gmg-is.co.uk/home; 2022. Accessed 21/4/22.

## BURNOUT

In 2022 there was a 29% shortfall in IR consultants with the UK workforce growing by only 2%. A more concerning statistic is the decreasing age of IR consultants leaving the workforce with the median age dropping from 55 in 2018 to 44 in 2022. Only 48% of trusts can provide adequate 24/7 IR services which places increasing strain on this already stretched workforce (1). A recent survey of UK IRs has shown moderate to severe scores of emotional exhaustion in 65% of respondents. The weekly hours worked and covering IR on call rotas were significant predictors. The major contributors of burnout were found to be increasing IR workload, shortage of IR colleagues and supporting staff (2, 3).

The provision of IR services out-of-hours is arduous for staff. Provision should be made within departments for rest time after busy on call. No clinician should be working when in a sleep deprived state. The recent Provision of Vascular Services document recommended that consideration should be given to reducing or stopping on-call commitments as surgeons enter the latter stages of their career (4). Although this may not be feasible because of local IR consultant staffing levels, a reasonable aspiration would be for IR consultants to either come off the on-call rota, or reduce their on-call commitment in their late 50s. From October 2023 it was possible for consultants over the age of 55 to take part or all of their pension benefits without having to leave their current job (partial retirement). This is an important mechanism that should be considered to allow our most senior and experienced IRs to continue to support the IR service.

**References**

20.1.: Royal College of Radiologists Census 2023.
20.2.: Al Rekabi A, Chen M, Patel N, Morgan R, McCafferty I, Haslam P, Hamady M. Well-being and Burnout Amongst Interventional Radiologists in the United Kingdom. Cardiovasc Intervent Radiol. 2023 Aug;46(8):1053–1063.
20.3.: Uberoi, R., Ramsden, W. & Halliday, K. Commentary on: Well-Being and Burnout Amongst Interventional Radiologists in the UK. *Cardiovasc Intervent Radiol* (2023).
20.4.: Provision of vascular Services https://www.vascularsociety.org.uk/_userfiles/pages/files/Resources/FINAL%20POVS.pdf

## SUMMARY

Interventional radiology is an essential service in all hospitals in the United Kingdom. Interventional radiology units vary in size depending on the size of the hospital, the services provided by the hospital and their proximity to other units. All patients should have access to emergency interventional radiology procedures when they require them. This will require a network arrangement between hospitals in some cases.

A wide number of clinical conditions are treated by interventional radiologists and the number of procedures grows year on year. Some basic interventional radiology procedures can also be performed by appropriately trained allied health professionals.

Paediatric interventional radiology is underserved by specialists in the UK and the increase in demand causes pressures on the interventional radiology workforce that must be met by workforce planning and by trusts going forward. Women should be encouraged to pursue a career in IR. Mitigations are in place to counter concerns about radiation exposure and family commitments.

There should be a formal method of assessment for IR trainees at completion of IR training before they commence consultant practice. The European Board of Interventional Radiology (EBIR) examination could be adopted by the UK to mandate that all IR trainees take this examination. A national IR registry is required to provide individual, trust and national data on UK IR practice.

Although interventional radiology is a subspecialty of radiology under the umbrella of the Royal College of Radiologists, this arrangement does not provide all the needs required by interventional radiology. A change of this model to an interventional radiology specialty or faculty within the RCR would provide substantial benefits for interventional radiology not least an increased knowledge of the IR workforce and control regarding how to increase it to meet the demands of patients.

Interventional radiologists must embrace clinical practice and aspire to take primary responsibility for patient care including a share in decision-making and IR clinics.

Finally, research is essential for the development of new treatments and the assessment of existing ones to assess their efficacy in comparison with other therapeutic options. Interventional radiologists should engage in research wherever feasible, encourage a research ethos in trainees under their tutelage and support the institution of increased designated interventional radiology academic positions nationally.

